# The Therapeutic Role of Perampanel in Treating Pediatric Patients With Dravet Syndrome: A Scoping Review

**DOI:** 10.7759/cureus.65017

**Published:** 2024-07-20

**Authors:** Anas S Alyazidi, Osama Y Muthaffar, Ahmed K Bamaga, Noura A AlAtwi, Suzan A Alshihri, Maram A Aljezani

**Affiliations:** 1 Medicine, Faculty of Medicine, King Abdulaziz University, Jeddah, SAU; 2 Pediatrics, King Abdulaziz University, Jeddah, SAU; 3 Pediatrics, East Jeddah General Hospital, Jeddah, SAU; 4 Pediatric Neurology, King Abdulaziz University Hospital, Jeddah, SAU; 5 Pediatric Neurology, King Fahad Medical City, Riyadh, SAU

**Keywords:** evidence-based, pediatric, seizure, scn1a, anti-seizure drug, epilepsy, perampanel, sodium channels, dravet syndrome

## Abstract

Sodium channelopathies are genetic disorders caused by mutations in genes, including sodium voltage-gated channel alpha subunit 1 (*SCN1A)*, that lead to several epilepsy syndromes. Traditional treatments with sodium channel blockers often have limited effectiveness and side effects. Dravet syndrome (DS), a severe epilepsy starting in infancy, presents significant treatment challenges. Perampanel (PER), a noncompetitive α-amino-3-hydroxy-5-methyl-4-isoxazolepropionic acid (AMPA) receptor antagonist, has shown promise for DS, reducing seizure frequency and improving quality of life (QoL). The limited availability of randomized controlled trials on PER among DS is challenging, but broader studies on refractory epilepsies offer insights. Real-world studies support PER's efficacy, underscoring its potential for managing refractory seizures in DS. Studies showed long-term effectiveness in reducing seizure frequency and enhancing QoL. While PER has minimal impact on cognitive development, it significantly improves seizure control. Numerous studies confirm the use of PER as an effective adjunctive treatment for DS; however, it is crucial to observe the safety profile, especially for pediatric sodium channelopathy patients. Common side effects include dizziness, drowsiness, and irritability, necessitating careful management. Long-term safety is generally favorable, but monitoring for behavioral and mood changes is essential. Additionally, the response to PER in DS varies widely, complicating its use. The limited clinical data and the need for careful dosage monitoring, especially in children, present significant challenges. Side effects, potential drug interactions, and high costs further complicate treatment. Despite increasing attention to its cost-effectiveness, accessibility remains limited in some regions, posing significant barriers for many families. In this paper, we review the role of PER in treating pediatric patients with DS, emphasizing clinical evidence and practical considerations.

## Introduction and background

Sodium channelopathies are genetic disorders caused by pathogenic variants in the voltage-gated sodium channel gene family, such as the sodium voltage-gated channel alpha subunit 1 (*SCN1A)*, *SCN2A*, *SCN4A*, and others [1]. These mutations can result in various syndromes, including epilepsy, episodic ataxia, and myotonia congenita, by increasing the excitability of neurons or muscles [1,2]. Current treatment approaches often involve the enhancement of GABAergic transmission in inhibitory interneurons that come into agreement with the reduced GABAergic tone in *SCN1A*-related seizures [3]. Meanwhile, sodium channel blockers like carbamazepine, phenytoin, and lamotrigine can be contraindicated in DS, as well as in other *SCN1A*-related seizure disorders [3,4]. Therefore, there is a growing need for alternative therapeutic options. Dravet syndrome (DS) is a severe type of epilepsy that starts during infancy [5]. It is characterized by frequent and prolonged seizures, posing a significant risk of mortality [5]. This condition presents substantial challenges for patients and their families due to its difficult-to-treat nature and associated developmental delays and cognitive impairments [6]. Furthermore, epidemiological data shows that it can affect 1 in 16,000-40,000 live births [6,7]. The management of DS typically involves a combination of drugs tailored to each individual's response, and ongoing research aims to identify effective therapies for better patient outcomes [7]. Moreover, patients with DS present with an increased mutation severity compared to other sodium channelopathies [8].

Perampanel (PER), a medication that acts as a non-competitive antagonist of the α-amino-3-hydroxy-5-methyl-4-isoxazolepropionic acid (AMPA) receptor on postsynaptic neurons, has emerged as a potential treatment option for DS [9]. Originally approved for partial-onset seizures and primary generalized tonic-clonic seizures in epilepsy patients, PER has shown promise in the pediatric population with DS due to its unique mechanism of action that sets it apart from other anti-seizure medications (ASM) [10]. Various clinical studies and case reports have investigated the effectiveness and safety of PER in children with DS, indicating that it could reduce seizure frequency and enhance the quality of life (QoL) for some patients [11,12].

However, like any ASM, the use of PER in pediatric patients requires careful consideration of potential side effects, such as dizziness, drowsiness, and behavioral changes, which can affect adherence and overall treatment effectiveness [13,14]. Therefore, continuous research and post-marketing surveillance are crucial for fully understanding the benefits and risks associated with PER in this vulnerable population.

This paper aims to provide a comprehensive overview of the role of PER in managing pediatric patients with DS. It will highlight the current clinical evidence, potential mechanisms of action, and considerations for its use in clinical practice.

## Review

Methodology

Review Protocol

**Figure 1:**
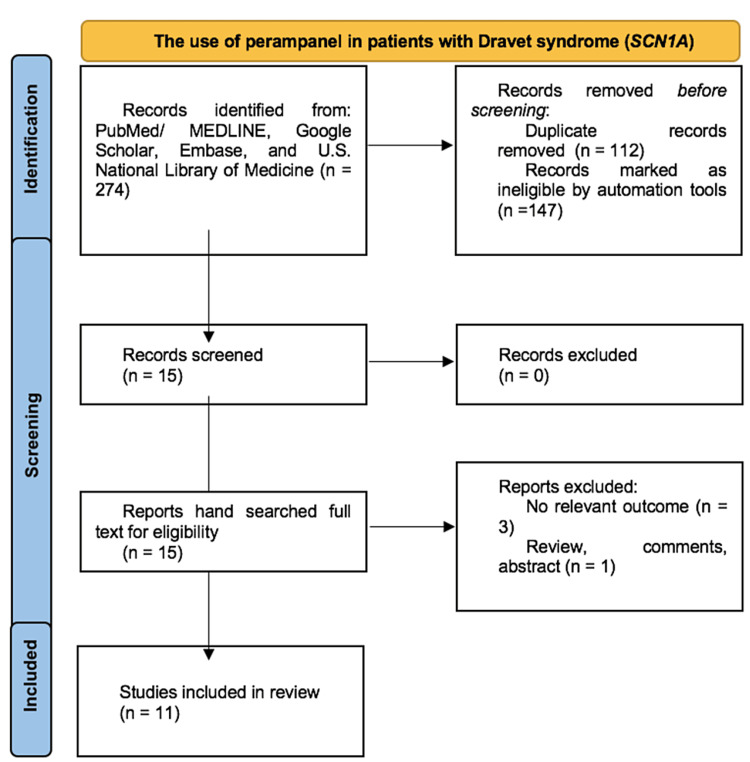
Flowchart of data retrieval. [15]

A review of the literature on the use of PER among patients with DS/*SCN1A* mutation was conducted by three researchers who independently and blindly searched the following electronic databases: PubMed/MEDLINE, Google Scholar, and Embase using the following terms: "Dravet syndrome", "*SCN1A*", "sodium channelopathies", and "Perampanel". Interventional and observational studies and clinical trials, which analyzed the outcome of PER among patients with confirmed DS/*SCN1A* diagnosis were included. Clinical trials were included using the U.S. National Library of Medicine's database, and those who met the inclusion criteria were thoroughly reviewed and analyzed (Figure 1).

Inclusion Criteria

Included studies were selected according to the following criteria: peer-reviewed articles, articles written in English, studies conducted on patients less than 18 years old (children), articles indexed in PubMed/MEDLINE, Google Scholar, and Embase, clinical trials indexed in the U.S. National Library of Medicine, and articles relevant to treatment and intervention assessing short- and long-term outcomes.

Exclusion Criteria

The exclusion criteria were structured to ensure that the review focused on relevant, up-to-date, peer-reviewed studies and clinical trials primarily targeting patients with DS. Non-English materials, irrelevant studies, outdated trials, and duplicates were excluded. Even after a thorough examination, studies that did not meet our inclusion criteria were excluded. In cases where multiple articles or trials were reported on the same survey, only the most comprehensive and recent publication was included, while duplicates were excluded. Furthermore, studies conducted solely on animal models without clinical relevance to human patients were excluded.

Subject Population

It included studies and clinical trials that addressed patients 18 years old and younger, both male and female.

Outcome Measures

The primary outcome measure was the reduction in seizure frequency from baseline. Furthermore, to assess the short- and long-term outcomes of the intervention based on clinical progression and reported side effects. Secondary outcomes included analyzing responder rates (≥50% reduction in seizure frequency), changes in seizure severity, and QoL assessments via validated measures as well as to assess logistical and economical aspects of the medication.

PER in treating sodium channelopathies

The main mechanism of action of PER centers around the inhibition of AMPA receptors, which are ionotropic receptors responsible for rapid synaptic transmission in the central nervous system (CNS) [16]. AMPA receptors, a subtype of glutamate receptors, play a crucial role in mediating fast excitatory neurotransmission and are pivotal in synaptic plasticity, which is essential for learning and memory processes. These receptors are permeable to sodium (Na+) and, to a lesser extent, calcium (Ca2+) ions, and their activation leads to depolarization of the postsynaptic membrane [16]. By obstructing AMPA receptors, PER diminishes excitatory postsynaptic potentials, resulting in a decrease in neuronal excitability [16,17]. This inhibition helps to balance the excitatory and inhibitory signals within the CNS, potentially reducing the hyperexcitability associated with various neurological disorders, such as epilepsy [16,17]. Additionally, the modulation of AMPA receptors by PER could influence synaptic plasticity, thereby affecting cognitive functions and potentially offering therapeutic benefits beyond seizure control [17,18]. Overall, the inhibition of AMPA receptors by PER is a critical mechanism that underlies its therapeutic effects in treating conditions characterized by excessive neuronal activity such as sodium channelopathies [18]. While PER has been extensively studied in the context of epilepsy, its use in sodium channelopathies is still emerging [18]. Several case reports and small-scale studies have suggested that PER may have benefits in conditions characterized by excessive neuronal excitability, including DS and other epilepsy syndromes associated with sodium channel mutations [19,20]. A notable study conducted by Miao et al. (2023) examined the effectiveness of PER in patients with genetic epilepsies [21]. The study demonstrated that PER significantly reduced the frequency of seizures in a subgroup of patients, indicating its potential usefulness in managing sodium channelopathies [21]. The *SCN1A* gene along with other seizure-causing genes which include G Protein Subunit Alpha O1 (*GNAO1)*, phosphatidylinositol glycan anchor (*PIGA)*, protocadherin 19 (*PCDH19)*, synaptic Ras GTPase activating protein 1 (*SYNGAP1)*, polymerase γ-1 (*POLG1)*, *POLG2*, and neuraminidase 1 (*NEU1)* are high responders to PER [11]. However, in the context of sodium channelopathy mutations, that is, *SCN1A*, *SCN2A*, *SCN8A*, sodium voltage-gated channel beta subunit 1 (*SCN1B)*, *SCN2B*, *SCN3A*, and *SCN9A*, only *SCN1A*-, *SCN2A*-, and *SCN8A*-related epilepsy, which encodes the alpha subunit of the voltage-gated sodium channel NaV1.1, NaV1.2, and NaV1.6, respectively, had emerging evidence suggesting effectiveness for PER in reducing seizure frequency. Nonetheless, despite the clinical similarity in patients with *SCN1A*, *SCN2A*, and *SCN8A* mutations, a study by Miao et al. demonstrated higher responses to PER significant statistical *p*-value in patients with the *SCN1A* than those with other ion channel genes, namely, *SCN2A* and *SCN8A* [21]. Moreover, anecdotal evidence and case reports have indicated potential improvements in patients with other sodium channel-related disorders, such as *SCN8A*-related epilepsy [22].

Efficacy assessment

The limited availability of randomized controlled trials (RCTs) specifically focused on PER use in DS remains a challenge. However, broader studies examining refractory epilepsies, including subgroups with DS, offer valuable insights. One such phase III double-blind, placebo-controlled trial conducted by French et al. evaluated PER as an adjunctive therapy for patients with uncontrolled partial-onset seizures [23]. Although not exclusively targeting DS, the study demonstrated significant reductions in seizures in the treatment group, indicating potential efficacy for DS [23]. Open-label studies have also contributed to understanding PER's effectiveness in DS. Furthermore, Nissenkorn et al. (2023) conducted a retrospective multicenter study that included pediatric patients with *SCN1A*-related developmental and epileptic encephalopathy, including DS. The study reported that 11 out of the 17 (64.7%) confirmed patients with DS due to *SCN1A* mutation were responders to PER, of those, 35.3% had >90% seizure reduction [11]. The overall reduction in seizure frequency for DS patients was 57.94%, and 35.29% [11]. Additionally, looking into other sodium channelopathies, a patient with *SCN2A* experienced a moderate improvement (50% reduction) while two patients with *SCN8A* demonstrated a 70% improvement in seizure frequency. The study suggests that PER could be an effective therapeutic option for DS, especially in *SCN1A* mutation [11]. Real-world evidence derived from multicenter observational studies adds to the understanding of PER's utilization in everyday clinical practice. Chang et al. (2020) conducted a single-center retrospective observational study that included three patients with DS treated with PER, reporting high seizure reduction rates (2/3 patients, 67%) [24]. In a different study, 4/5 patients reached 50% seizure reduction and two experiencing complete seizure freedom [14]. Moreover, PER efficacy has also been described as disease-specific in DS [24]. Additionally, a study showing the efficacy of PER in a newborn with early myoclonic epilepsy with *SCN1A* mutation suggests PER is *SCN1A*-mutation-specific [25]. These findings emphasize the potential of PER in managing refractory seizures in patients with DS and across the age group spectrum in the pediatric population.

Long-term effectiveness and outcome

Seizure Frequency and Severity

**Figure 2:**
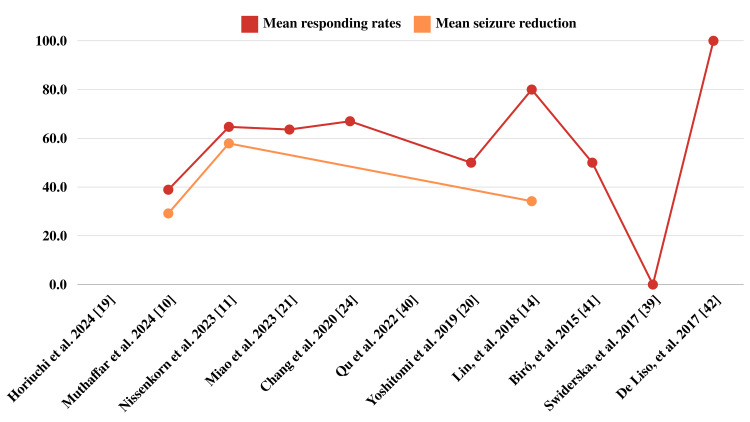
Mean response rates and seizure reduction. Image credit: Anas S. Alyazidi.

Multiple studies have provided evidence of PER's long-term effectiveness in reducing seizure frequency among patients with DS. According to Chang et al., patients with DS showed a high level of long-term efficacy for PER. In particular, over a prolonged period of evaluation, a total of 67% of patients with DS experienced positive treatment outcomes and achieved adequate seizure freedom. These results imply that the drug may successfully maintain seizure control and enhance the general clinical results for patients with DS [24]. A report by Turón-Viñas et al. demonstrated the high effectiveness of PER in a child that achieved complete seizure remission within weeks of medication initiation and no reported side effects [26]. In terms of seizure frequency reduction, lower pharmacological response to PER is substantially correlated with developmental delay. Typically, children with more profound developmental delays and drug-resistant seizures have a more severe genetic insult (Figure 2) [21].

Cognitive Development

The impact of PER on cognitive development in patients with DS is of significant interest. While controlling seizures is crucial, long-term cognitive outcomes are equally important. Studies on the long-term effects of PER on adolescent cognition have shown that, aside from attention, PER had no discernible effects on cognitive parameters [27]. The topic is of importance in the context of DS as a genotype-phenotype relation has been proposed associating early onset of seizures and worse cognitive outcomes in the condition [28-31]. A systematic review associated a neutral cognitive profile with PER reporting no systematic cognitive deteriorations or improvements in the use of this medication [32].

Quality of Life

Enhancing the QoL for patients with DS and their families is a primary objective of treatment. A study showed that DS can present higher levels of comorbidities and lower QoL [33,34]. However, studies assessing QoL among patients with DS after initiating QoL are ultra-rare. Therefore it is important to look into studies assessing QoL levels before and after the initiation of PER in patients with refractory epilepsies. A study in Melbourne, Australia assessed the QoL using the Quality of Life in Epilepsy-Patient-Weighted (QOLIE-10-P) scale and reported improved QoL among highly refractory patients after administrating PER as late add-on adjunctive therapy [35]. However, a study assessed the QoL using the Pittsburgh Sleep Quality Index and observed improvement in the index's performance a month after the use of PER [12]. Moreover, long-term studies have demonstrated that PER treatment is associated with improvements in QoL measures [36]. Nonetheless, improvements in QoL are crucial for the overall well-being of patients with DS and their families.

PER as an adjuvant medication

Numerous studies have confirmed the effectiveness of PER as an adjunctive treatment in reducing seizure frequency in patients with DS. A pivotal study conducted by Gao et al. assessed the efficacy, safety, and tolerability of PER as an adjunctive treatment. The study included 12 patients with DS, their response rate to the medication was 50.0% and the seizure-free rate reached 25.0% [37]. This is compared to two other studies in which responding percentages were 67.0% and 80.0% in patients with DS [14,24]. Nonetheless, the study by Gao et al. provided strong evidence to support the use of PER as an adjunctive treatment [37]. Moreover, Ishikawa et al. described a patient presenting with early myoclonic encephalopathy due to *SCN1A* mutation that was successfully treated with PER as an adjunctive treatment [25]. Another study by Zhang et al. included three patients diagnosed with DS describing the patients' attaining a response rate of 66.7% [38]. It suggests an effective role for the treatment especially among certain subtypes of focal epilepsies [38]. Additional research is required to assess the long-term effectiveness and safety of PER as an adjunctive treatment.

Adverse events and safety profile

Considering the frequently intricate co-morbidities linked to sodium channelopathies, the safety profile of PER is a crucial factor to consider. It is associated with common side effects such as dizziness, drowsiness, and irritability, which require careful management, particularly in pediatric populations [10]. However, the noncompetitive nature of PER's antagonistic action may provide a more favorable side effect profile when compared to conventional sodium channel blockers. The long-term safety profile of PER in pediatric patients is generally favorable, although certain adverse effects require attention. Common side effects include dizziness, drowsiness, irritability, and ataxia [10,13]. Moreover, Most adverse effects are mild to moderate in severity and can be managed by adjusting the dosage. However, careful monitoring is necessary, particularly over extended treatment periods, due to the potential for behavioral and mood changes [13]. In the study of Chang et al., the long-term safety of PER was also reviewed. Not merely specific to DS but for the cohort as a whole; adverse events were reported in 21% of the patients that varied from emotional problems, fatigue, nasal bleeding, and skin allergy and led to the discontinuation of the drug among four patients in a month [24]. Echoing Chang et al., Lin et al. also reported skin manifestations in addition to irritability, dizziness, and somnolence [14]. PER is generally well-tolerated in pediatric patients, although such adverse effects have been documented. Overall, the most common side effects were mental issues coupled with physical manifestations. While long-term safety data are reassuring, ongoing vigilance is essential to identify any rare or delayed adverse effects. This includes harmful adverse events such as suicidal thoughts, which were reported by Swiderska et al. [39]. Nonetheless, studies reporting a lack of adverse events for PER continue to exist [26]. In other studies, the incidence of adverse events is possible with a lack of serious adverse events (Table 1) [37].

**Table 1:** Evidence-based summary of literature studies. PER, perampanel; DS, Dravet syndrome; *SCN1A*, sodium voltage-gated channel alpha subunit 1

#	Author	Date	Study design	Sample size	Number of patients with >50% efficacy	Mean responding rates	Adverse events	Conclusion
1	Horiuchi et al. [19]	2024	Observational, case report	1	1	N/A	Mild	The report indicated seizure reduction after switching the patient to PER mainly due to the role of the previous medication in inducing seizure, however, PER also played a significant role in controlling the patient's status epilepticus.
2	Muthaffar et al. [10]	2024	Observational, retrospective	18	7	38.9% (mean seizure reduction of 29.17%)	Mild	The study revealed a significant relationship between younger patients and increased effectiveness of PER. The dosage of PER also had some impact on its efficacy. Overall, the study provided evidence of PER's potential as a promising therapy for certain patients with DS.
3	Nissenkorn et al. [11]	2023	Observational, retrospective	17	11 (6 patients had >90% reduction in seizure)	64.7% (mean seizure reduction of 57.9%)	Moderate	The study suggested an effective therapeutic option for PER in children and adults with rare genetic epilepsies, especially in certain genetic subgroups including SCN1A.
4	Miao et al. [21]	2023	Observational, retrospective	13	7	63.6%	Mild	Patients with the *SCN1A* demonstrated better response to PER compared to other sodium channelopathies (including *SCN2A* and *SCN8A*). Clinical features, such as the age of seizure onset and developmental delays, had no statistical significance.
5	Chang et al. [24]	2020	Observational, retrospective	3	2	67%	Mild	The study concluded a significant good short-term and long-term efficacy in patients with DS.
6	Qu et al. [40]	2022	Observational, prospective	15	N/A	High	Mild	The response rates to PER in DS were high.
7	Yoshitomi et al. [20]	2019	Observational, retrospective	10	5	50%	Mild	PER may be promising in certain patients with DS.
8	Lin, et al. [14]	2018	Observational, retrospective	5	4	80% (mean seizure reduction of all participants 34.2%)	Mild	The study suggested that PER was conspicuously efficacious in patients with DS.
9	Biró, et al. [41]	2015	Observational, retrospective	2	1	50%	Mild	The study provided data on the potential effectiveness of PER in children with refractory epilepsies
10	Swiderska, et al. [39]	2017	Prospective and retrospective	1	Discontinued early (at 3 months) due to lack of seizure control	0%	Moderate	PER showed to be a broad-spectrum medication that works well for patients experiencing both focal and generalized seizures.
11	De Liso, et al. [42]	2017	Observational retrospective	1	1	100%	Mild	The study suggested that PER had a considerable impact on the treatment of adolescents with epilepsy.

PER compared to other ASMs

According to the available literature, PER may not always be more effective than other well-established treatments, even though it can help some DS patients experience fewer seizures overall [26]. For example, stiripentol has shown a strong reduction in the frequency of seizures, especially when combined with clobazam and valproate [43]. Likewise, cannabidiol (CBD) has demonstrated great potential and frequently leads to a significant decrease in convulsive seizures [44]. Moreover, two double-blind clinical trials demonstrated efficacy for the use of CBD for patients with DS [45]. Furthermore, PER has a notable side effect profile as described in this review. These adverse effects can be particularly challenging in the pediatric population typical of DS. In contrast, stiripentol and CBD generally have more manageable side effect profiles, although they are not without their risks [46,47]. Also, PER’s metabolism via CYP3A4 introduces potential for drug-drug interactions, particularly in patients on polypharmacy [48]. Stiripentol, while also having significant interactions due to its inhibition of CYP3A4 and CYP2C19, has a well-established protocol for managing these interactions [49]. CBD, metabolized by CYP3A4 and CYP2C19, also requires careful monitoring but generally has fewer severe interactions [50]. Therefore, given the current literature, PER, despite its effectiveness in many reported studies, does not necessarily appear to be more effective than other ASMs in treating seizures in DS. While PER can be beneficial for some patients, its variable efficacy and notable side effect profile suggest that it may not be superior to other established treatments.

Challenges and limitations in the use of PER

**Figure 3:**
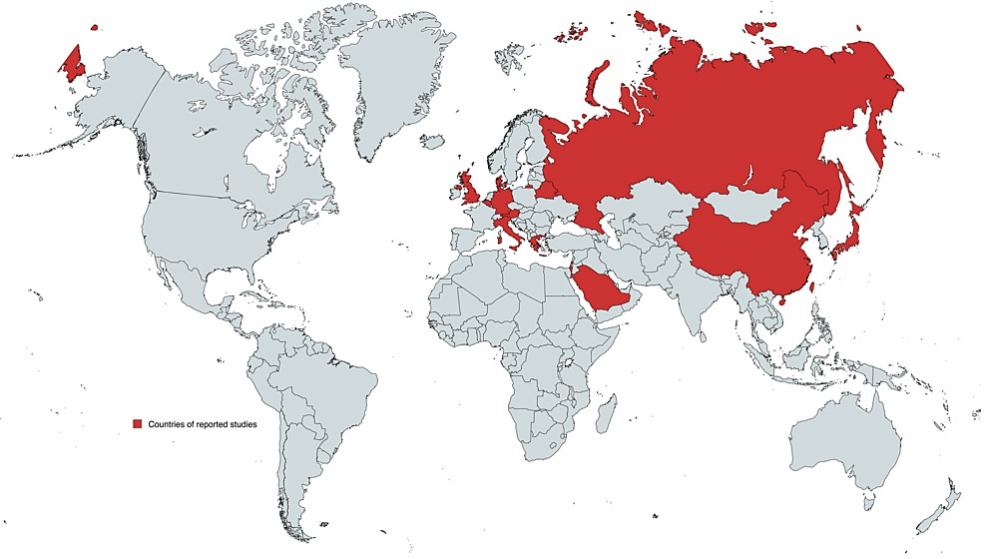
Countries reporting studies on the use of perampanel in Dravet syndrome. Image credit: Anas S. Alyazidi/MapChart.

The response to PER in patients with DS can vary greatly. While some patients may experience a notable decrease in seizures, others may see little or no improvement [14,24,26,37]. This variability complicates the decision-making process for doctors considering PER as a treatment option. Furthermore, there is limited clinical data available specifically evaluating the effectiveness of PER in treating DS, especially in long-term settings. Most studies have focused on other types of epilepsy, leaving a gap in well-founded guidelines for its use in DS [42]. Also, the potential interactions of PER in DS have the potential to affect the effectiveness and safety of the medications [48]. Furthermore, determining the optimal dosage of PER can be difficult. The drug has a narrow therapeutic range, and finding the right dose that effectively reduces seizures while minimizing side effects requires careful monitoring [51,52]. This task is especially challenging in pediatric populations, where dosing must be adjusted based on weight and age. Moreover, ensuring strict adherence to dosing schedules can be a hurdle, particularly in children with developmental delays and cognitive impairments commonly seen in DS [53]. Caregivers may face significant efforts to maintain compliance, adding to their burden. Moreover, the cost of PER is high, making it a significant barrier for many families. Insurance coverage may not fully cover the medication, resulting in substantial out-of-pocket expenses [54]. This financial burden can be especially challenging considering the long-term nature of epilepsy treatment. However, recent studies are suggesting the affordability of PER and has been receiving increasing attention due to its cost-effectiveness results, especially compared to other ASM maintenance therapies [55,56]. Additionally, access to newer ASMs like PER can be limited in certain regions, restricting treatment options for patients with DS. This limitation is particularly notable in low-resource settings where healthcare systems may be slow to adopt newer therapies (Figure 3).

## Conclusions

We analyzed molecular and real-world data on the use of PER in clinical practices, revealing its potential as a therapeutic choice for sodium channelopathies through AMPA receptor antagonism. While existing evidence is promising, additional research is necessary to thoroughly determine its effectiveness and safety in this context. As our understanding of sodium channelopathies and the pharmacodynamics of PER advances, this treatment has the potential to become a fundamental approach to managing these complex disorders.

PER exhibits significant potential as a long-term supplementary treatment for pediatric patients diagnosed with DS, particularly those with confirmed *SCN1A* mutations. The enduring decrease in seizure frequency, along with potential enhancements in cognitive development and QoL, underscores its importance in clinical practice. Although it is generally safe and well-tolerated, continuous monitoring and additional research are crucial for optimizing its usage and comprehensively understanding its long-term effects on individuals with DS. Exploring combination therapies involving PER with traditional ASMs may offer synergistic advantages in managing these conditions.
